# Supplementary material for: Neuroprotective Effects of Transferrin in Experimental Glaucoma Models
Source: Int J Mol Sci. 2022 Oct 22;23(21):12753. doi: 10.3390/ijms232112753 (PMC9659282; doi:10.3390/ijms232112753)
Supplement: Supplementary file 1 [file ijms-23-12753-s001.zip › ijms-1953638-supplementary.pdf]

Supplementary Material

Supplementary Materials and Methods

NMDA-mediated excitotoxicity and $CoCl_2$-mediated hypoxia on explants

Explants from adult male Wistar rats were subjected to 100, 300 or 500 µM NMDA or $CoCl_2$ for 24 hours, washed and maintained in culture in completed neurobasal medium until 96 hours (n = 2-3 explants per condition). RGCs and photoreceptors (PR) immunostaining was then performed on flat-mounted retinal explants using a goat anti-rat Brn3a (1:300, Santa Cruz) and an anti-Peanut Agglutinin conjugated with fluorescein isothiocyanate (1:200; Merck) primary antibodies, respectively. For each explant, 3 to 5 standardized photomicrographs similarly distributed for all conditions to cover central to peripheral retina were taken at 40X magnification under fluorescence microscope. Quantification of Brn3a-positive RGCs and peanut agglutinin-positive PRs was performed then performed on each image by an operator masked for the treatment. The number of cells was reported as the mean number of cells per field (photomicrograph).

Legend of Supplementary Figures

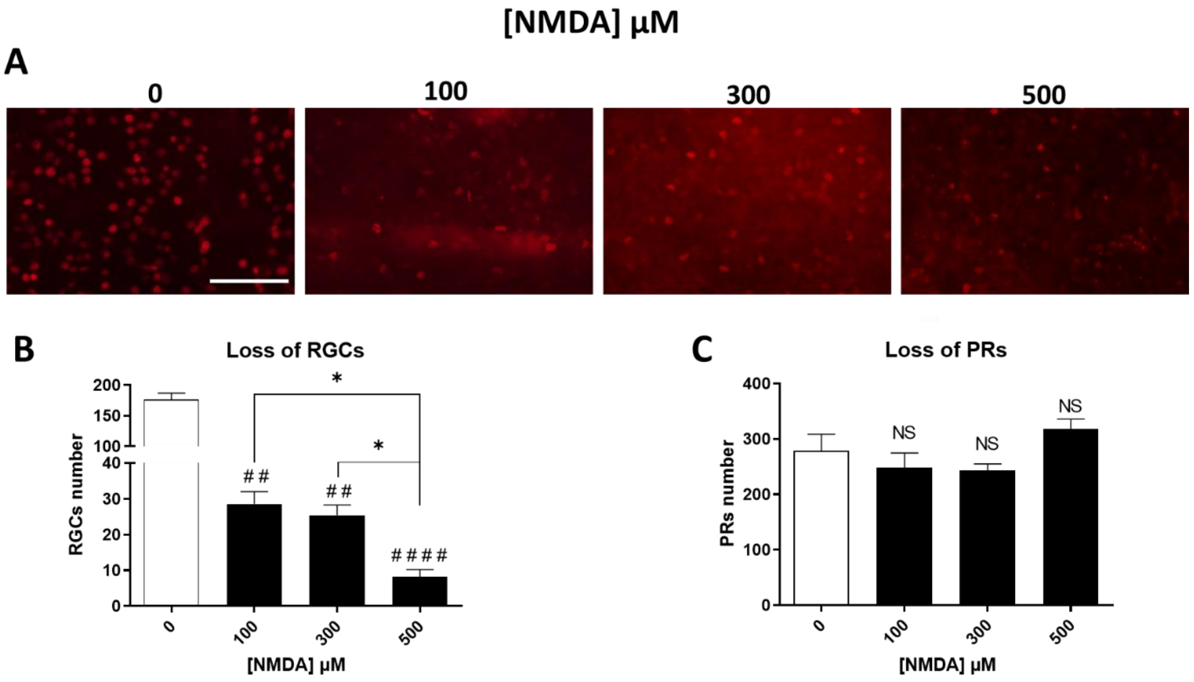

Supplementary Figure S1. NMDA induces RGC loss in rat retinal explant. (A) Representative images of retinal whole-mounts prepared for Brn3a immunostaining following 24 h incubation with 100, 300 or 500 µM NMDA and further cultured for 72 h (96 h total). Untreated explant served as control. (B) NMDA induces a dose-dependent loss in Brn3a positive cells. (C) NMDA does not affect the survival of photoreceptors. Data represents means ± SEM, n = 2-3 explants per condition. Statistical analysis was performed with Kruskal-Wallis with Dunn's test for multiple comparisons. ## $p < 0.01$, #### $p < 0.0001$ compared to control; * $p < 0.05$; ns, not significant. Scale bar: 100 µm

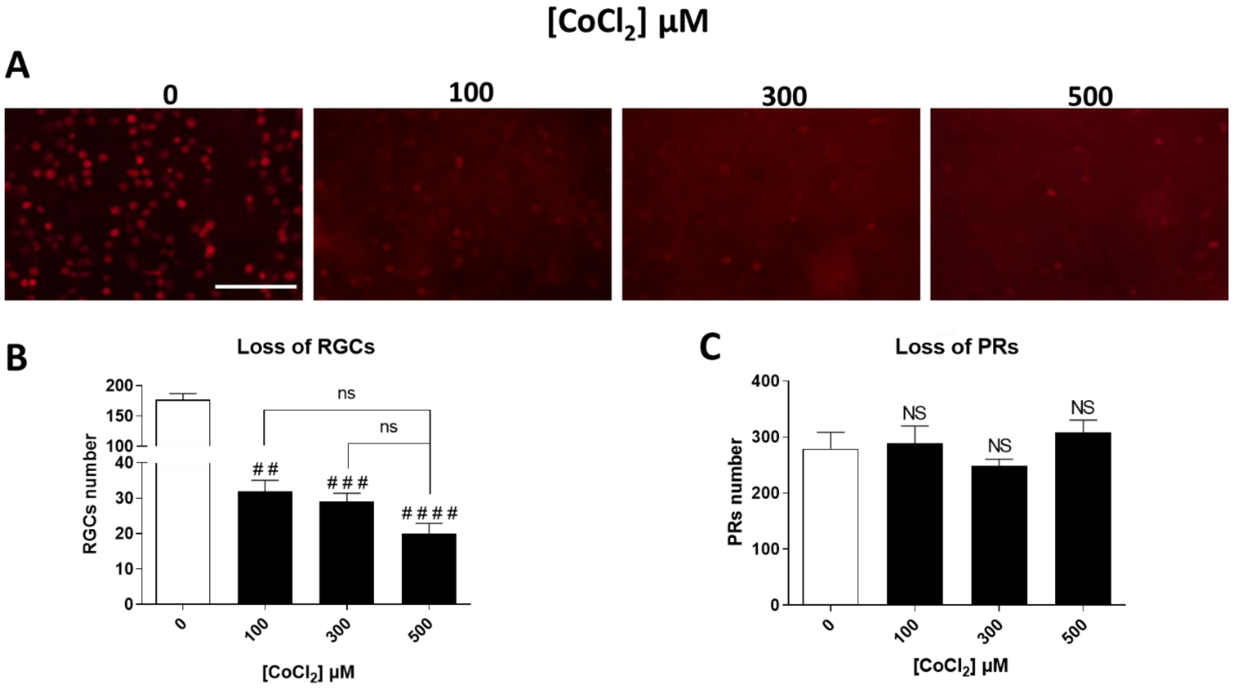

Supplementary Figure S2. $CoCl_2$ induces RGC loss in rat retinal explant. (A) Representative images of retinal whole-mounts prepared for Brn3a immunostaining following 24 h incubation with 100, 300 and 500 µM $CoCl_2$ and further cultured for 72 h (96 h total). Untreated explant served as control. (B) $CoCl_2$ induces a dose-dependent loss in Brn3a positive cells. (C) $CoCl_2$ does not affect the survival of photoreceptors. Data represents means ± SEM, n = 2-4 explants per condition. Statistical analysis was performed with Kruskal-Wallis with Dunn's test for multiple comparisons. ## $p < 0.01$; ### $p < 0.001$; #### $p < 0.0001$ compared to control; ns, not significant. Scale bar: 100 µm

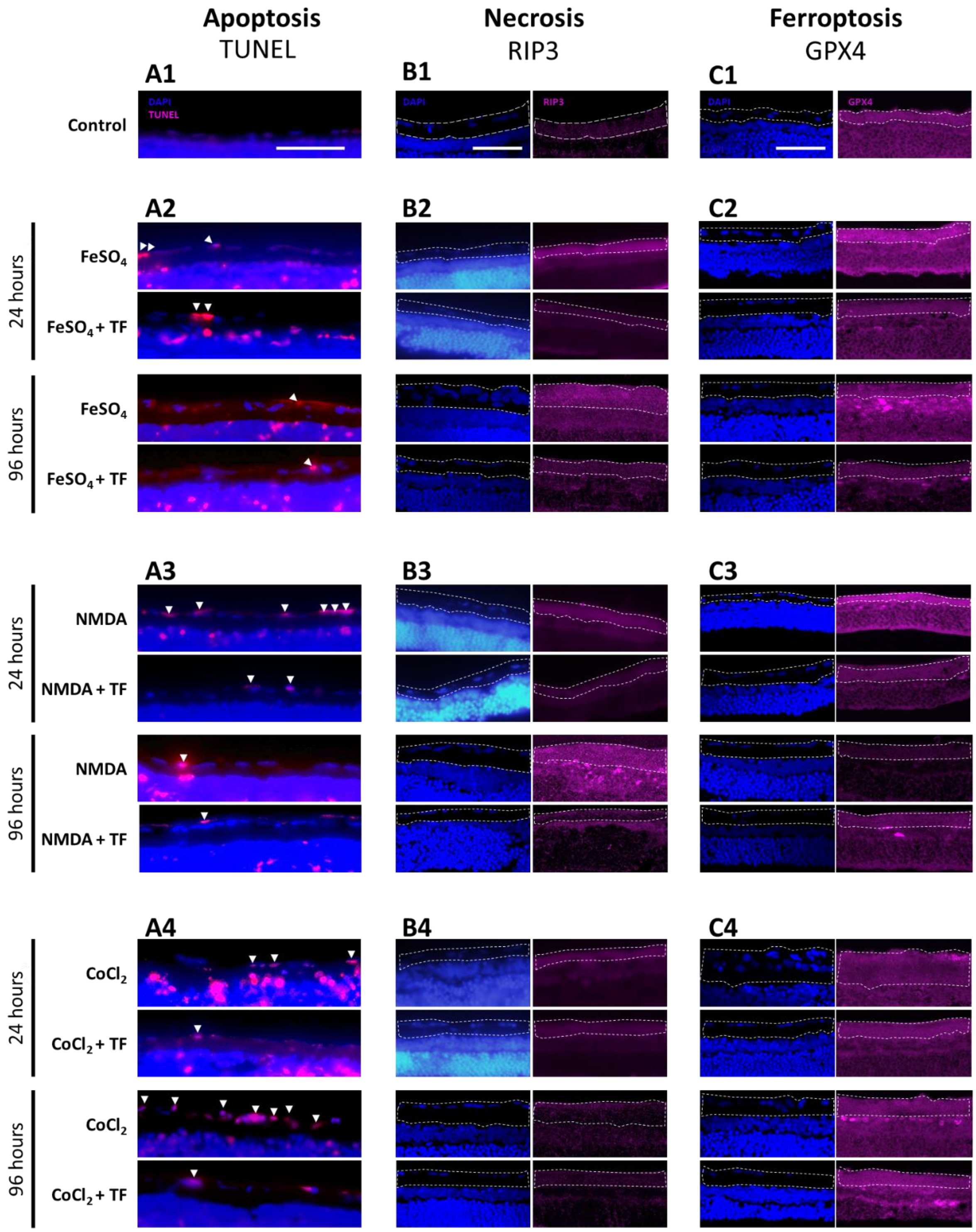

Supplementary Figure S3. Transferrin protects against different cell death mechanisms. Representative images of the activation of apoptosis, necrosis or ferroptosis in *ex vivo* models exposed to $FeSO_4$ (A2-C2), NMDA (A3-C3) and $CoCl_2$ (A4-C4) in presence or in absence of TF monitored at 24 and 96 hours, by respectively, the quantification of TUNEL-positive RGCs

(A - White arrow), and by the change in RIP3 (B) or GPX4 (C) immunostaining intensity in RGC layer (outlining in white dots) compared to controls (A1-C1). Scale Bar: 50 µm.

Supplementary Tables

Supplementary Table S1. Average baseline IOP by group

	All animals	Naïve Control (normotensive)	Negative Control (BSS)	Experimental Group (TF)
Baseline IOP (mm Hg)	13.8 ± 0.2	14.2 ± 1.7	13.5 ± 1.7	14.5 ± 1.7
p-value[a]		-	0.9917	0.9937

IOP was measured using a rebound tonometer on vigil animals. Baseline IOP was measured over three consecutive days and the average IOP across all three days was considered as baseline value for randomization. Each group had an IOP average that was not statistically different from any other group. Values are means ± SEM; [a] Two-way ANOVA followed by Tukey's test for multiple comparisons; compared Naïve control group.
BSS, Balanced Salt Solution; TF, human apo-Transferrin.

Supplementary Table S2. Effect of OHT on IOP over the experimental period

Groups	Day 2	Week 1	Week 2	Week 3	Week 4	p-value
Naïve Control (normotensive)	13.53 ± 0.27	12.78 ± 0.26	11.7 ± 0.50	12.23 ± 0.52	12.04 ± 0.13	< 0.0001
Negative Control (BSS)	21.70 ± 2.38	18.06 ± 0.56	19.08± 0.41	21.27 ± 1.16	20.78 ± 0.87	< 0.0001
Experimental Group (TF)	19.67 ± 0.74	18.84 ± 0.75	19.64 ± 0.48	20.06 ± 0.48	20.64 ± 0.77	< 0.0001

IOP was measured 48h after OHT induction, then weekly thereafter. All OHT groups had significantly higher IOP than the naïve control group beginning at 48h after OHT. The experimental group (TF) and negative control (vehicle) group IOPs were not statistically different as determined by repeated measures ANOVA with Tukey's test for multiple comparisons over the experimental period. Values are means ± SEM. BSS, Balanced Salt Solution; TF, human apo-Transferrin.
